# Safety of antithrombotic therapy in subjects with hereditary hemorrhagic telangiectasia: prospective data from a multidisciplinary working group

**DOI:** 10.1186/s13023-019-1278-z

**Published:** 2019-12-26

**Authors:** Eleonora Gaetani, Fabiana Agostini, Angelo Porfidia, Igor Giarretta, Daniela Feliciani, Luigi Di Martino, Annalisa Tortora, Antonio Gasbarrini, Roberto Pola, on behalf of the Multidisciplinary Gemelli Group for HHT, Giulio Passali, MariaElena Riccioni, Alfredo Puca, Carmelo Sturiale, Laura Riccardi, Carmine Di Stasi, Andrea Contegiacomo, Anna Emilia Del Ciello, Manuel Ferraro, Anna Franca Cavaliere, Emanuela Lucci Cordisco, Giuseppe Zampino, Valentina Giorgio, Veronica Ojetti, Giuseppe Marrone, Gabriele Spoletini, Gabriella Locorotondo, Gaetano Lanza, Erica De Candia, Elisabetta Peppucci, Luigi Corina, Maria Teresa Lombardi

**Affiliations:** 1 Multidisciplinary Gemelli Group for HHT, Fondazione Policlinico Universitario A. Gemelli IRCCS, Rome, Italy; 2 Division of Internal Medicine and Gastroenterology, Fondazione Policlinico Universitario A. Gemelli IRCCS, Rome, Italy; 3 Department of Medicine, Fondazione Policlinico Universitario A. Gemelli IRCCS, Università Cattolica del Sacro Cuore, L.go A. Gemelli 8, 00168 Rome, Italy; 4 Division of Internal Medicine, Fondazione Policlinico Universitario A. Gemelli IRCCS, Rome, Italy

## Abstract

Subjects with the rare autosomal dominant disease Hereditary Hemorrhagic Telangiectasia (HHT) may develop medical conditions that require antithrombotic therapy (AT). However, safety of AT is uncertain in these patients and the only data currently available derive from retrospective analyses of registries and/or databases. At the HHT Centre of the ‘Fondazione Policlinico Universitario A. Gemelli IRCCS’ (Rome, Italy), a prospective study is currently ongoing to evaluate the safety of AT in subjects affected by HHT. The study is enrolling subjects with a definite diagnosis of HHT who receive an AT prescription by one of the physicians of the HHT Centre. The primary outcome is the number of hemorrhagic events, distinguished in major, clinically relevant non-major (CRNM), and minor bleedings, according to the criteria of the International Society on Thrombosis and Hemostasis (ISTH). Another primary outcome is worsening of epistaxis upon initiation of AT, assessed using the internationally accepted Epistaxis Severity Score (ESS). Additional outcomes are changes in hemoglobin levels and changes in the need of blood transfusion after initiation of AT. Here, we present the results of an interim analysis, conducted on the 12 HHT subjects that have been enrolled so far. After a mean follow-up of 6.5 ± 0.8 months, no major bleedings, no CRNM bleedings, and no minor bleedings different from epistaxis were recorded. Worsening of epistaxis upon initiation of AT was documented only in one patient, but did not require discontinuation of AT. There were no significant changes in the mean ESS measured before and after initiation of AT. There were no significant changes in hemoglobin levels and need for blood transfusion after initiation of AT. Although preliminary, these are the first prospective data on the safety of AT in HHT patients. Our interim analysis suggests that, when prescribed by experienced physicians in a multidisciplinary setting, AT is well tolerated by HHT patients. More patients and a longer follow-up are needed to confirm these findings.

**Dear Editor**,

We read with interest the studies published by Shovlin and colleagues [1] and by Riera-Mestre and colleagues [2] in two recent issues of the Orphanet Journal of Rare Diseases. Both studies dealt with the issue of thrombotic diseases in subjects with Hereditary Hemorrhagic Telangiectasia (HHT) and the lack of reliable data on the safety of antithrombotic therapy (AT) in these patients. Shovlin and colleagues carried out a retrospective audit across the eight HHT Centres of the European Reference Network for Rare Multisystemic Vascular Diseases and assessed the safety of direct oral anticoagulants (DOACs) in 28 HHT patients who were prescribed by other clinicians a total of 32 treatment cycles for either atrial fibrillation (AF) or venous thromboembolism (VTE). They found a worsening of epistaxis in 24/32 treatment cycles, with 11 treatment discontinuations. Riera-Mestre and colleagues analysed the RIETE Registry to assess the clinical characteristics, therapeutic approaches and clinical outcomes during the course of anticoagulant therapy in 23 patients with HHT affected by VTE. They found that the use of anticoagulants may increase the risk for bleeding, but barely influences mortality, since most bleeds came from nasal or digestive telangiectasia and not from large HHT-related vascular malformations. These studies are valuable, but both have the limitation of being retrospective analyses of databases and/or registries, as acknowledged by the same authors, who admit that prospective studies are needed to establish whether HHT patients may tolerate AT.

At the HHT Centre of the ‘Fondazione Policlinico Universitario A. Gemelli IRCCS’ (Rome, Italy), we are currently conducting a prospective study whose objective is the safety of AT in subjects with HHT. The inclusion criteria are a ‘definite’ diagnosis of HHT, a new prescription of AT, willingness to participate to the study, and the signature of informed consent. As established in the literature [3], the diagnosis of HHT is considered ‘definite’ when the following criteria are fulfilled: genetic confirmation of the disease and/or presence of at least 3 of the following 4 Curaçao criteria: 1) recurrent and spontaneous nosebleeds (epistaxis); 2) multiple telangiectases on the skin of the hands, lips or face, or inside of the nose or mouth; 3) arteriovenous malformation (AVMs) or telangiectases in one or more internal organs, including the lungs, brain, liver, intestine, stomach, and spinal cord; 4) a family history of HHT (i.e. first-degree relative certainly affected). For new prescription of AT, we intend that patients have not been on any AT at least for the last 3 months and, at the time of enrolment, receive a new prescription of an anticoagulant or an antiplatelet medication by one of our physicians, for an established medical reason. Upon enrolment, participating subjects undergo a detailed medical interview and their clinical history is carefully reviewed by the study investigators, in order to define the phenotypical features of the HHT disease in terms of family history, presence/absence of epistaxis, presence/absence of AVMs in various organs and tissues, history of gastrointestinal (GI) bleeding, anemia, need for blood transfusion. The severity of epistaxis is assessed at the time of enrolment, before initiating AT, using the internationally accepted Epistaxis Severity Score (ESS) [4]. Hemoglobin levels are measured at the time of enrolment, before initiating AT. The need of blood transfusion in the 3 months before the initiation of AT is assessed at enrolment by retrospective analysis of clinical histories and medical data. The primary outcome of the study is the number of hemorrhagic events occurring during AT. Events are classified according to the criteria of the International Society on Thrombosis and Hemostasis (ISTH) [5, 6], as follows: i) major bleedings were defined as fatal bleedings, or symptomatic bleedings in critical areas or organs (such as intracranial, intraspinal, intraocular, retroperitoneal, intraarticular or pericardial, or intramuscular with compartment syndrome), or bleedings causing a fall in hemoglobin level of 2 g/dL or more, or leading to transfusion of two or more units of whole blood or red cells; ii) clinically relevant non-major (CRNM) bleedings were defined as acute or subacute clinically overt bleedings that did not satisfy the criteria for major bleedings but led to hospital admission for bleeding, or physician-guided medical or surgical treatment for bleeding, or a change in AT due to bleeding; iii) minor bleedings, with the exception of epistaxis, were defined as acute clinically overt bleedings that did not meet the criteria for either major or CRNM bleedings. Another primary outcome of the study is the worsening of epistaxis upon initiation of AT. In any individual, this is defined as a change of category (from mild to moderate/severe or from moderate to severe) in the ESS measured during the course of AT. A comparison is also performed between the mean ESS measured at enrolment and after at least 3 months of AT. Two additional outcomes are: changes in hemoglobin levels, measured after at least 3 months of AT, compared to hemoglobin levels measured at enrolment; changes in the need of blood transfusion, determined after at least 3 months of AT, compared to the needs displayed by patients in the 3 months before the initiation of therapy. Follow-up visits are scheduled 14 days, 1 month, and 3 months after enrolment, and every 3 months thereafter. Each outcome event is adjudicated by the study investigators. To detect possible unreported events, symptom questionnaires are administered to patients at the time of any follow-up visit and hospitalization reports are scrutinized for unreported outcomes, in case patients were hospitalized between visits. The SPSS 20.0 and GraphPad Prism 7.0 software is used to perform statistical analysis. Descriptive statistics are used to outline patients characteristics. Results are expressed as mean ± standard deviation (SD) or number and percentage. For parametric variables, we compare means using paired samples Student’s t test. For non-parametric variables, we use Wilcoxon Signed Rank Test for paired samples. *P* values less than 0.05 are considered statistically significant.

Here, we present the results of an interim analysis, conducted on 12 patients, who are those with at least 3 months of follow-up at the time of the analysis. At enrolment, patients had the characteristics presented in Table 1. They were mostly males (66.6%), with a mean age of 63.9 ± 13.4 years. They all had chronic epistaxis (100.0%). The mean ESS was 3.9 ± 2.8. Eleven patients (91.6%) presented mucocutaneous telangiectases at sites other than endonasal. A positive family history of HHT was present in 11 patients (91.6%). Pulmonary AVMs were present in 5 subjects, on a total of 10 who were screened by computed tomography (CT) scan of the chest. Hepatic AVMs were present in 1 patient, on a total of 10 who were screened by either ultrasound, CT scan, and/or magnetic resonance imaging (MRI) of the abdomen. GI AVMs were present in 5 patients, on a total of 7 who were screened by endoscopic procedures. There were no patients with AVMs of the central nervous system (CNS), on a total of 7 who were screened by either CT scan and/or MRI of the brain and spinal cord. History of GI bleeding was present in 5 subjects (41.6%), who were the same 5 with a diagnosis of GI AVMs. Six patients (50.0%) had anemia (defined as hemoglobin < 13.0 g/dl for men and < 12.0 g/dl for women, as established by the World Health Organization) in the 3 months before the initiation of AT. The mean hemoglobin level was 11.4 ± 2.9 g/dl. There was 1 patient (8.3%) that had required blood transfusion in the 3 months before the initiation of AT. The indications for anticoagulant therapy were AF (4 patients) and VTE (1 patient). These 5 patients were prescribed with vitamin K antagonists (1 patient) and DOACs (4 patients). The indications for antiplatelet therapy were previous stroke/transient ischemic attack (5 patients) and previous myocardial infarction (2 patients). These 7 patients were prescribed acetylsalicylic acid (5 patients) and clopidogrel (2 patients). Mean follow-up was 6.5 ± 0.8 months, with 6.2 ± 1.1 months for anticoagulant therapies and 6.9 ± 0.4 months for antiplatelet therapies.

**Table 1:** Characteristics of the population

Mean age (years±SD)	63.9 ± 13.4
Gender (male/female ratio)	8/4
Epistaxis (n/total)	12/12
ESS at enrolment (mean ± SD)	3.9 ± 2.8
Mucocutaneous teleangiectases other than endonasal (n/total)	11/12
Anemia in the 3 months before enrolment (n/total)	6/12
Mean Hb at enrolment (g/dl ± SD)	11.4 ± 2.9
Blood transfusion(s) in the 3 months before enrolment (n/total)	1/12
Family history of HHT (n/total)	11/12
Pulmonary AVMs (n/screened)	5/10
Hepatic AVMs (n/screened)	1/10
Cerebral AVMs (n/screened)	0/7
Gastrointestinal AVMs (n/screened)	5/7
Previous gastrointestinal bleeding (n/total)	5/12
*Indication to AT*	12/12
Atrial Fibrillation (n/total)	4/12
Venous thromboembolism (n/total)	1/12
Previous stroke/transient ischemic attack (n/total)	5/12
Previous myocardial infarction (n/total)	2/12
*Number of AT courses / number of patients*	
Anticoagulant therapy (n/total)	5/12
Antiplatelet therapy (n/total)	7/12
*Mean duration of follow-up (months ± SD)*	6.5 ± 0.8
Mean duration of antiplatelet therapy courses (months±SD)	6.9 ± 0.4
Mean duration of anticoagulant therapy courses (months±SD)	6.2 ± 1.1

Safety outcomes are presented in Table 2 and Fig. 1. No major bleedings and no CRNM bleedings were recorded. There were no minor bleedings different from epistaxis. Worsening of epistaxis was recorded in 1 patient, who was taking acetylsalicylic acid. The mean ESS measured after at least 3 months of AT was not statistically different from that measured at the time of enrolment, before the initiation of AT (3.2 ± 2.6 vs 3.9 ± 2.8 respectively, 95% IC − 3.2-1.5, *p* = 0.2988). There were no significant changes in mean hemoglobin levels measured after at least 3 months of AT, compared with those measured at the time of enrolment, before the initiation of AT (11.5 ± 2.5 vs 11.4 ± 2.9 g/dL, 95% CI − 1.5-1.5, *p* = 0.9609). There were no changes in the need for blood transfusion upon initiation of AT. None of the 12 patients required termination of AT during the follow-up period.

**Table 2:** Safety outcomes

Major bleedings (n/total)	0/12
CRNM bleedings (n/total)	0/12
Minor bleedings different from epistaxis (n/total)	0/12
Worsening of Epistaxis (n/total)	1/12
ESS at enrolment vs during AT (mean ± SD)	3.9 ± 2.8 vs 3.2 ± 2.6 (95% IC −3.2-1.5, *p* = 0.2988)
Mean Hb at enrolment vs during AT (g/dl ± SD)	11.4 ± 2.9 vs 11.5 ± 2.5 (95% CI −1.5-1.5, *p* = 0.9609)
Patients needing transfusions prior to AT vs during AT (n/total)	1/12 vs 0/12
Discontinuation of AT (n/total)	0/12

**Fig. 1:**
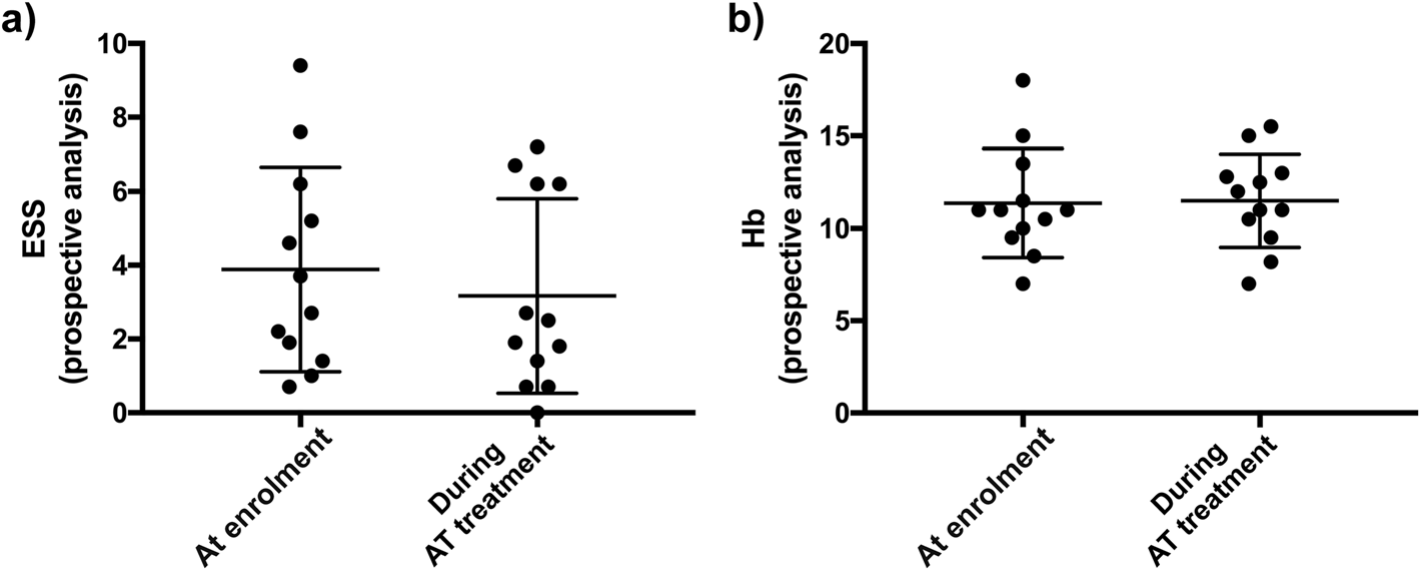
Impact of antithrombotic therapy (AT) on the severity of epistaxis and hemoglobin (Hb) levels. The severity of epistaxis, assessed using the Epistaxis Severity Score (ESS), was similar at enrolment (before starting AT) and after at least 3 months of AT (**a**). Hb levels were similar at enrolment and after at least 3 months of AT (**b**)

This is the first prospective study evaluating the safety of AT in subjects with HHT. Our interim results indicate that, when prescribed by experienced physicians in the context of a multidisciplinary working group, AT is well tolerated by HHT patients. One strength of this study is that all patients had a ‘definite’ diagnosis of HHT, different from other studies in the literature. For instance, there is a report by Devlin and colleagues in which the study population consisted of subjects who participated to an online survey and were considered affected by HHT based on their answers to a questionnaire [7]. Also in the study of Riera-Mestre and colleagues the authors were not able to retrospectively assess Curaçao criteria and/or collect genetic data for all patients [2]. Another strength of our study is that all patients were at high hemorrhagic risk. Indeed, 100% of them had epistaxis, 40% had history of GI bleeding, about 50% had anemia before initiating AT, and about 40% presented visceral AVMs. This is different from the populations analysed by other studies. For instance, in the study of Edwards and colleagues, subjects with substantially lower rates of epistaxis (75%), GI bleeding (23%), anemia (35%), and visceral AVMs (10%) were enrolled [8]. An additional strength of our study is that all AT prescriptions were personally made by physicians with multiannual experience in the field of thrombosis, cardiovascular diseases, and AT. Therefore, we are certain that all patients had an established indication to AT and were prescribed the right type of medication at the right dosage. This was not warranted in the recent report of Shovlin and colleagues, in which treatment was initiated by other clinicians in diverse external institutions [1]. Finally, the prospective nature of our study allowed us to assess in an objective manner not only the number of bleedings, but also their severity, with the possibility to distinguish between major, CRNM, and minor bleedings, as established by the ISTH [5, 6]. Also changes in the severity of epistaxis, hemoglobin levels, and need of blood transfusion were objectively evaluated.

This study has some limitations. The sample size is relatively small. However, HHT is a rare disease and the number of HHT patients that require AT is consequently limited. Another potential limitation of our study is that patients with more severe HHT and therefore higher risk for bleeding might have been denied AT, thus generating a selection bias. However, until now, only one of the HHT patients that we screened was denied AT. This was a subject with AF, who also had advanced liver cirrhosis (Child-Pugh C), spontaneously elevated international normalized ratio (INR), and low platelet count. Therefore, the decision not to prescribe anticoagulant therapy was not based on the severity of HHT itself, but to the presence of concomitant diseases that are universally considered a contraindication to AT. Finally, due to the small sample size, our study does not allow distinction, in terms of safety, between antiplatelet and anticoagulant therapies and between different antiplatelets and different anticoagulants. Nonetheless, it is interesting to note that, among the 4 patients taking DOACs, there were no cases of bleeding except for the pre-existing epistaxis, which however did not worsen in any patient during follow-up observation. This is different from what was found by Shovlin and colleagues in their recent audit (13 cases in which epistaxis worsened on a total of 32 treatment cycles wit DOACs, whit 11 cases of treatment discontinuation and 8 cases of extreme bleeding response). This difference may be due to casualty, considering the small number of patients investigated both in our and Shovlin’s studies, but might also depend on the fact that, as mentioned above, our prescriptions were always made by physicians experienced in thrombosis and anticoagulation in a multidisciplinary setting dedicated to the cure of HHT. This might have led to prescriptions tailored on each individual patient, always taking into consideration parameters such as age, body weight, renal function, history of GI bleeding, number and type of visceral AVMs, concomitant diseases and possible pharmacological interactions, which are crucial when AT is prescribed.

In conclusion, the interim results of our prospective study provide preliminary evidence that HHT patients tolerate AT, when it is prescribed by experienced physicians in the context of a multidisciplinary setting. The completion of this study, with the inclusion of more patients and a longer follow-up, is needed to confirm these findings.

## Data Availability

The datasets used and analysed during the current study are available from the corresponding author on reasonable request.

## Abbreviations

AF: Atrial fibrillation
AT: Antithrombotic therapy
AVM: Arteriovenous malformation
CRNM: Clinically relevant non-major
CT: Computerized tomography
DOAC: Direct oral anticoagulant
ESS: Epistaxis Severity Score
GI: Gastrointestinal
HHT: Hereditary hemorrhagic telangiectasia
INR: International normalized ratio
ISTH: International Society on Thrombosis and Hemostasis
MRI: Magnetic resonance imaging
SD: Standard deviation
VTE: Venous thromboembolism
